# The macrophage infectivity potentiator of *Trypanosoma cruzi* induces innate IFN-γ and TNF-α production by human neonatal and adult blood cells through TLR2/1 and TLR4

**DOI:** 10.3389/fimmu.2023.1180900

**Published:** 2023-05-26

**Authors:** Sarra Ait Djebbara, Saria Mcheik, Pauline Percier, Noria Segueni, Antoine Poncelet, Carine Truyens

**Affiliations:** ^1^ Laboratory of Parasitology, Faculty of Medicine, and ULB Center for Research in Immunology (UCRI), Université Libre de Bruxelles (ULB), Brussels, Belgium; ^2^ Service Immune Response, Sciensano, Brussels, Belgium

**Keywords:** IFN-γ, TNF-α, TLR2, TLR4, adjuvant, neonatal immunity, macrophage infectivity potentiator (Mip), *Trypanosoma cruzi*

## Abstract

We previously identified the recombinant (r) macrophage (M) infectivity (I) potentiator (P) of the protozoan parasite *Trypanosoma cruzi* (Tc) (rTcMIP) as an immuno-stimulatory protein that induces the release of IFN-γ, CCL2 and CCL3 by human cord blood cells. These cytokines and chemokines are important to direct a type 1 adaptive immune response. rTcMIP also increased the Ab response and favored the production of the Th1-related isotype IgG2a in mouse models of neonatal vaccination, indicating that rTcMIP could be used as a vaccine adjuvant to enhance T and B cell responses. In the present study, we used cord and adult blood cells, and isolated NK cells and human monocytes to investigate the pathways and to decipher the mechanism of action of the recombinant rTcMIP. We found that rTcMIP engaged TLR1/2 and TLR4 independently of CD14 and activated the MyD88, but not the TRIF, pathway to induce IFN-γ production by IL-15-primed NK cells, and TNF-α secretion by monocytes and myeloid dendritic cells. Our results also indicated that TNF-α boosted IFN-γ expression. Though cord blood cells displayed lower responses than adult cells, our results allow to consider rTcMIP as a potential pro-type 1 adjuvant that might be associated to vaccines administered in early life or later.

## 1 Introduction

Vaccines aim to prevent morbidity and mortality due to infectious diseases by triggering a long-lasting protective memory immune response against pathogens before their natural encounter. Vaccination is one of the most effective public health interventions to reduce the burden of infectious diseases (1). However, owing to inherent differences of the immune system between neonates/infants and adults, it is generally more difficult to induce protective immune responses in early life. It relates to, among others, an inherent skewing to anti-inflammatory innate responses and to Th2 and regulatory T cell adaptive responses at the expense of the Th1 response in early life (2–5). Additionally, neonatal T cells seem less prone than adult ones at forming memory (6). These features represent a drawback to protect infants as soon as possible by vaccination. Nonetheless, some vaccines given at birth or in the first months of life, like vaccines against tuberculosis, hepatitis B or poliomyelitis, provide rapid protection, either because their efficacy relies mostly on neutralizing antibodies preventing the entry and the replication of viruses, and less on T cell responses, or because they induce a high initial inflammatory response able to overcome the difficulty in early life to mount type 1 immune responses (6, 7). It is indeed well recognized that the innate response shapes the adaptive response, regulating its orientation toward type 1, type 2 or other types of T cell responses adapted to efficiently fight the type of pathogen, and controls the development of memory T cells (8, 9).

There is an imperative need to improve vaccine efficacy in early life to still reduce the life cost of infectious diseases in children, predominantly in the first months of life. One of the key elements is to optimize and develop new adjuvants adapted for neonatal immunization, particularly for enhancing type 1 immune responses (1, 7). Based on our observation that *T. cruzi* maternal or congenital infection boosted type 1 responses to vaccines given to infants in the first months of life (10), we have identified a protein of this protozoan parasite (Tc) able to induce rapid and robust IFN-γ production by cord blood cells, the macrophage (M) infectivity (I) potentiator (P) (TcMIP) (11). This protein had previously been described as a virulence factor secreted by the parasite, which favors its entry and survival into host cells (12, 13). We thus recently disclosed another property of this protein, i.e., its ability to immuno-stimulate human blood cells. We also showed that the recombinant TcMIP (rTcMIP) increased antibody responses in mouse models of neonatal vaccination against ovalbumin and pertussis, skewing the response towards higher production of the Th1-related isotype IgG2a (11). These results supported that rTcMIP might be a candidate adjuvant for vaccines administered in early life. In this study, we have analyzed further the effects of rTcMIP and its mechanism of action on neonatal and adult innate immune cells. We found that rTcMIP engaged TLR1/2 and TLR4 independently of CD14 and activated the MyD88 but not the TRIF signaling pathway. Through these receptors, rTcMIP induced the release of IFN-γ by cord blood IL-15-primed NK cells, and of TNF-α by monocytes and myeloid dendritic cells, which boosted the release of IFN-γ. Though the responses of neonatal cells were inferior to that of adult ones, it reinforces considering rTcMIP as a potential adjuvant for pediatric vaccines.

## 2 Material and methods

### 2.1 rTcMIP recombinant protein

The sequence of the macrophage infectivity potentiator of *T. cruzi* (rTcMIP – UniProtKB - Q09734) is published (12, 13). The recombinant macrophage infectivity potentiator of *T. cruzi* (rTcMIP) was produced as previously described (11). It is a fusion protein between TcMIP (~22kDa) and glutathione-S-transferase of *Schistosoma japonicum* (GST, ~26 kDa). The fusion protein is referred to as rTcMIP in the text. All experiments include the GST alone (#PK-RP577-1243-1, Bio-Connect B.V., Huissen, The Netherlands) as a control. Endotoxins present in the purified rTcMIP were eliminated as described in (11), till reaching levels < 0.5 EU/mg rTcMIP. The rTcMIP concentrations mainly used in this work are between 2.5 and 5 µg/mL, containing thus less than 0.005 EU/mL (i.e. 1 pg entotoxin/mL), which is well below the threshold of 0.5 EU/mL accepted by the FDA in medical devices (14) and endotoxin levels found in most commercialized vaccines (15).

### 2.2 Ethics statement

The study was conducted according to the principles expressed in the Declaration of Helsinki and was approved by the Ethical Committee Erasme-U.L.B. of the Faculty of Medicine, U.L.B. (protocols P2011-254 and P2014-339). Informed written consent was obtained from adult voluntary donors and from the parents of newborns to collect peripheral blood and umbilical cord blood respectively.

### 2.3 Blood collection

Umbilical cord blood was collected from healthy newborns at the delivery room of the Erasme hospital, Brussels, Belgium. Adult blood samples were collected from healthy volunteers aged between 20 and 60. Blood samples (10 mL) were drawn in endotoxin free sodium heparinized tubes (Vacutainer, BD Benelux N.V., Erembodegem, Belgium) and kept at room temperature before being processed within one (adult) or eight (cord) hours after collection.

### 2.4 Blood cells and cell lines

Cultures were performed either with whole blood 10-fold diluted in culture medium, with cord blood mononuclear cells (CBMCs) or peripheral blood mononuclear cells (PBMCs) isolated on NycoPrep 1.077 density gradient (#AXI-1114550, Axis-Shield, Oslo, Norway), or with blood NK cells purified by negative selection using MACS and the NK Cell Isolation Kit according to the manufacturer’s instructions (#130-092-657, Miltenyi Biotec B.V., Leiden, The Netherlands). Purity of CD3^-^ CD56^+^ NK cells was above 90% as assessed by FACS analysis. The human monocytic cell line THP-1 was kindly provided by Fabienne Willems (Institute for Medical Immunology, Faculty of Medicine, U.L.B., Gosselies, Belgium). HEK-Blue™ cells stably transfected with human TLR2 or TLR4 or untransfected (HEK-Blue Null) and transfected with the reported gene SEAP (secreted embryonic alkaline phosphatase) (#hkb-htlr2, hkb-htlr4, hkb-null1) were purchased from InvivoGen (Toulouse, France). To note that HEK293 cells transfected with TLR2 constitutively express TLR1 and TLR6 (and not TLR4) while cells transfected with TLR4 do not express TLR2. They neither expressed TLR3, TLR5, TLR7, TLR8 and TLR9.

### 2.5 Cell stimulation

Ten-fold diluted whole blood or 150,000 CBMCs, PBMCs, NK or THP-1 cells were seeded in 96-well U-bottom sterile, polystyrene culture plates (#650180, Greiner Bio One, Vilvoorde, Belgium) and cultured at 37°C in humidified atmosphere of 5% CO2 in RPMI 1640 medium with L-glutamine 2 mM and HEPES 25 mM (BE12-115F/12), non-essential amino acids 0.1 mM (#BE13-114E), Na pyruvate 1 mM (#BE13-115E), penicillin 100U/mL, streptomycin 100 µg/mL (all from Lonza, Verviers, Belgium) and fetal bovine serum 10% (#FBS-LE-12A, Capricorn Scientific, Ebsdorfergrund, Germany). The following stimulants were used, at concentrations indicated in the figures: rTcMIP, GST (#PK-RP577-1243-1, Bio-Connect B.V., Huissen, The Netherlands), lipopolysaccharide from *E. coli* 055:B5 (LPS-B5, #tlrl-pb5lps, InvivoGen), Pam3CSK4 (#tlrl-pms, InvivoGen), Pam2CSK4 (#tlrl-pm2s-1, InvivoGen), rhIL-2 (Sigma-Aldrich, Overijse, Belgium), IL-12 (#219-IL, R&D systems, Abingdon, UK), IL-15 (#247-IL, R&D Systems), IL-18 (#rcyec-hil18, InvivoGen). THP-1 cells were pretreated with phorbol myristate acetate (PMA) 500 nM (#tlrl-pma, InvivoGen) for 3h at 37°C. After 24h or 72h, cell viability evaluated by Trypan blue dye exclusion was always > 95%, supernatants were collected and stored at -80°C until cytokine measurements and cells were analyzed by flow cytometry for identification of IFN-γ and TNF-α producing cells. In some experiments, rTcMIP was pre-incubated at room temperature with FK506 (tacrolimus, #F4679, Sigma Aldrich) at the concentrations indicated before being added to cultures.

Reported HEK cells transfected with TLR2 or TLR4 or untransfected were cultured at a density of 2.8 x 10^5^ cells/mL and treated with the indicated stimuli for 24 h at 37°C in DMEM with glucose, glutamine and Na pyruvate (#BE.12.604F, Lonza) complemented with penicillin 100U/mL, streptomycin 100 µg/mL and fetal bovine serum 10%. Secreted alkaline phosphatase (SEAP) activity in supernatants was measured using QUANTI-Blue reagent (#rep-qbs, InvivoGen) according to the manufacturer’s instructions.

Endotoxin contamination has been ruled out in all reagents not announced to be endotoxin-free by the Pierce LAL test (#88282, Thermo Fisher Scientific, Belgium) and mycoplasma contamination has been ruled out in cell lines with the PCR Mycoplasma Test Kit (# A3744, PanReac AppliChem, Darmstadt, Germany). All procedures were performed under laminar flow with sterile endotoxin-free reagents and material.

### 2.6 Cytokine assays

IFN-γ and TNF-α levels in culture supernatants were determined by ELISA using CytoSet kits (#CHC1233 and #CHC1753 respectively, Thermo Fisher Scientific). Assays were performed in duplicate following the manufacturer’s instructions. Detection limits were 2 pg/mL.

### 2.7 Antibody neutralization assays

The following antibodies (Abs) were used (all from InvivoGen): anti-TNF-α (htnfa-mab1), anti-TLR1 (#mabg-htlr1), anti-TLR2 (#mab-mtlr2), anti-TLR4 (#mab2-htlr4), anti-TLR6 (#mabg-htlr6) or anti-CD14 (#mabg-hcd14). They were added to cell cultures at a concentration of 5 µg/mL 1h before rTcMIP stimulation. Mouse IgG matched isotype Abs (#mabg1-ctrlm) were used as a control.

### 2.8 NF-κB inhibition assays

Resveratrol (#tlrl-resv) and dexamethasone (#tlrl-dex) were purchased from InvivoGen. Resveratrol 80 µM or dexamethasone 40 ng/mL were added to cell cultures 1h before rTcMIP stimulation (at 5 µg/mL) for 24h. As concentrated stock solutions of these reagents were prepared in DMSO and ethanol respectively, diluted DMSO or ethanol was used as control. The reagents and the solvents had no effect on cell viability evaluated by the Trypan blue dye.

### 2.9 MyD88/TRIF inhibition assay

MyD88 and TRIF specific inhibitory peptides (Pepinh-MyD: #tlrl-pimyd and Pepinh-TRIF: #tlrl-pitrif) and the control peptide (Pepinh-Control) were purchased from InvivoGen. They were added at a concentration of 10 µM to cell cultures 6h before rTcMIP stimulation for 24h.

### 2.10 Intracellular cytokine staining and flow cytometry

Brefeldine A (#B7651, Sigma) was added at 5 µg/mL for all the culture duration or the last 24h of culture to block cytokine release. After each incubation time (4h, 24h or 72h), cells were harvested, washed with Stain Buffer (#554656, BD) and permeabilized with the Fixation/Permeabilization solution (#554714, BD) for 20 min at 4°C. Cells were then stained for 30 min at 4°C with a mix containing predetermined optimal concentrations of antibodies and FcR Blocking Reagent following manufacturer’s instructions (#130-059-901, Miltenyi) in Perm/Wash™ Buffer (#554714, BD). Cells were washed with the Perm/Wash buffer after each incubation time. Labelled cells were suspended in Stain Buffer before flow cytometry analysis. The antibodies directed against the following human markers, purchased from BD Biosciences unless otherwise indicated, were used: for intracellular IFN-γ detection: CD3-BV421 (#562426) or CD3-PerCP (#345766), CD56-FITC (#562794) or CD56-APC (555518), CD14-PE (#21620144x21/2, Immunotools, Friesoythe, Germany), CD16-APC (#21278166x2, Immunotools) or CD16-PE (555407), IFN-γ-AF700 (#557995) or IFN-γ FITC (340449); for intracellular TNF-α detection in monocytes and NK cells: CD3-FITC (#555916), CD14-APC-H7 (#560180), CD16-APC (#302012, BioLegend Europe BV, Amsterdam, The Netherlands), CD56-BV421 (#562751), HLA-DR-PE-Cy7 (#560651), TNF-α-PE (#559321); for TNF-α detection in dendritic cells (DCs): anti- Lineage Cocktail (CD3, CD14, CD19, CD20, CD56)-FITC, (#348701, BioLegend), CD34-FITC (#345801, used only on CBMCs), HLA-DR-PE-Cy7 (#560651), CD123-PerCPCy5.5 (#306016, BioLegend), CD11c-BV711 (#563130), TNF-α-PE (#559321). The gating strategy allowing to delineate the CD56^bright^ and CD56^dim^ subsets of NK cells is described in (16). The gating strategy to identify myeloid CD11c^+^ CD123^-^ and plasmacytoid CD11c^-^ CD123^+^ DCs is described in (17). Matching anti-mouse Ig isotypes were used as a control. Single stained Microbeads (#552843, BD Biosciences) were used for compensations. Data acquisition was performed on FACSCanto II or Fortessa X20 flow cytometers (Becton Dickinson, California, USA) with the FACSDivaTM software. Data were analyzed using FlowJo software V9.9.5 (Treestar, California, USA).

### 2.11 Expression of results and statistical analysis

Data are expressed as means ± standard error of the mean (SEM) or as Box and Whisker plots. Differences between groups were tested for significance using the Mann Whitney Wilcoxon test. Statistical significance was accepted if P was <0.05. Statistical analyses were performed with GraphPad Prism 6.07 (GraphPad Software, La Jolla, California, USA).

## 3 Results

### 3.1 rTcMIP stimulates cord blood CD56^bright^ NK cells to produce IFN-γ

**Figure 1:**
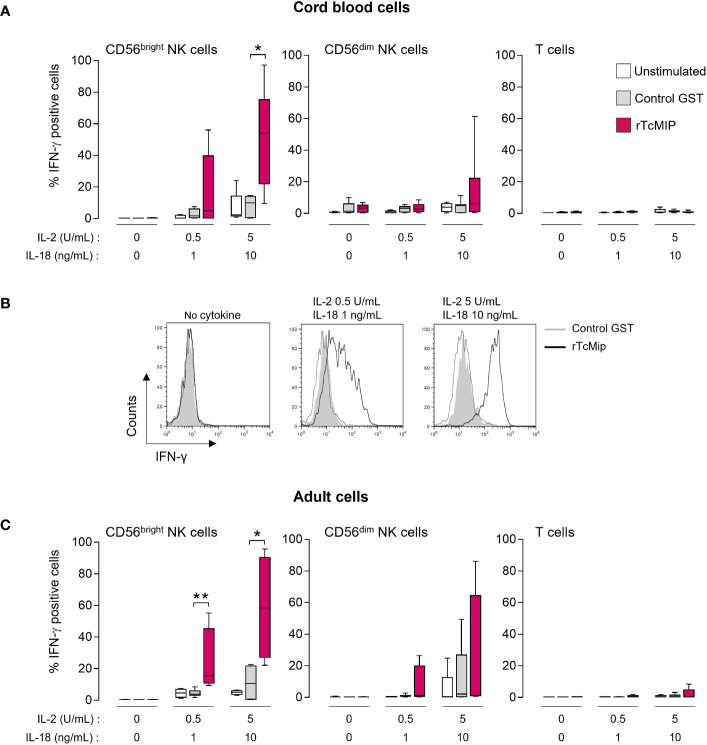
rTcMIP stimulates cord blood and adult blood NK cells to produce IFN-γ. Whole cord **(A, B)** or adult blood cells **(C)** were stimulated with rTcMip 2.5 μg/mL in the absence or presence of two concentrations of IL-2 (0.5 or 5 U/mL) combined to IL-18 (1 or 10 ng/mL) for 72h. GST 1.25 μg/mL alone was used as a control. The frequency of IFN-γ positive cells in CD56bright and CD56dim NK cells and T cells was determined by flow cytometry. Results are presented as mean ± SEM from **(A)** 5 cord blood samples and **(C)** 5 adult blood cells. **(B)** A representative histogram of the response of cord blood CD56bright NKcells is shown. *P ≤ 0.05, **P<0.05 as compared with cells cultured with GST (Mann Whitney Wilcoxon test).

To identify by flow cytometry the IFN-γ producing cells, whole cord blood cells were stimulated with rTcMIP in the presence of IL-2 and IL-18 as in the experiments allowing the identification of this protein (11). **Figure 1A, B** shows that IFN-γ was produced by NK cells and not T cells. Amongst the NK cells, the CD56^bright^ subset was the main source of IFN-γ. A low proportion of CD56^dim^ NK cells produced IFN-γ and T cells remained almost negative. No IFN-γ was detected in other blood cells (data not shown). The response of CD56^bright^ NK cells to rTcMIP increased with rising amounts of IL-2 and IL-18. At the highest concentrations of IL-2 and IL-18, all tested newborns and adults produced IFN-γ, which was not the case at lower IL-2 and IL-18 concentrations. Noteworthy, the proportions of responding CD56^bright^ NK cells from neonates and adults were similar (**Figure 1A** vs. **1C**) (mean ± SEM: 51.5 ± 12.7 vs. 58.6 ± 16.5% respectively, p=0.457). It is worth mentioning that in the absence of IL-2 and IL-18, we occasionally observed a low response by CD56^bright^ NK cells (detailed in **Supplementary Figure 1A**). When mononuclear neonatal (CBMCs) or adult (PBMCs) cells were used in place of whole blood samples, NK cells remained the only producers of IFN-γ in response to rTcMIP (**Supplementary Figure 1B**). All subsequent experiments were performed with mononuclear cells.

These results raise the question whether rTcMIP activates NK cells either directly and/or indirectly through signals delivered by accessory cells such as monocytes or dendritic cells.

### 3.2 IL-15 synergizes with rTcMIP to induce IFN-γ production by cord blood mononuclear cells

**Figure 2:**
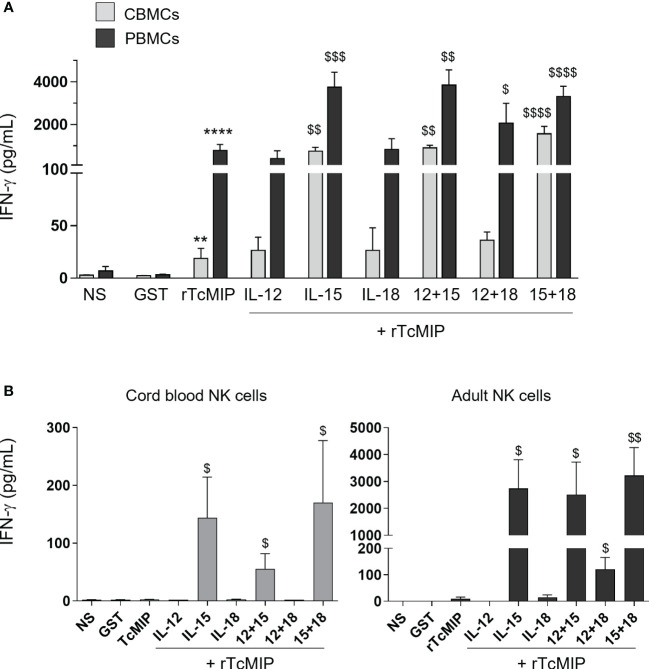
Effect of co-stimulatory cytokines on the rTcMIP-induced IFN-γ response of cord blood and adult mononuclear cells and purified NK cells. Mononuclear cells **(A)** or purified NK cells **(B)** were stimulated with rTcMIP (2.5 µg/mL) or GST as control (1.25 µg/mL) for 72h in the absence or presence of IL-12 (0.5 ng/mL), IL-15 (2.5 ng/mL) or IL-18 (0.25 ng/mL) or a combination of these cytokines as indicated, used at the same concentrations. Supernatants were assayed for IFN-γ secretion by ELISA. Results are expressed as mean ± SEM of 6 (with exogenous cytokines) to 20 (without cytokines) CBMCs or PBMCs, and of 3 (experiments with IL-12 or IL-18) to 6 (experiments with IL-15) purified NK cells samples. **: P<0.005, ****: P<0.0001 vs. GST. $: P<0.05, $$: P < 0.005, $$$: P < 0.005, $$$$: P< 0.0001 vs. rTcMIP alone (Mann Whitney Wilcoxon test). NS, non-stimulated cells.

We investigated the co-stimulatory effect of IL-15, IL-12 and IL-18, cytokines potentially produced by monocytes and dendritic cells and known to prime/co-stimulate NK cells (18, 19). We used cytokine concentrations that did not induce IFN-γ production when used alone (**Supplementary Figure 2**). The IFN-γ response of CBMCs and PBMCs to rTcMIP associated with these cytokines was measured by ELISA after 72h of culture (**Figure 2A**). We observed that rTcMIP induced IFN-γ production by CBMCs and PBMCs even in the absence of co-stimulatory cytokines, though the adult response to rTcMIP alone was markedly higher than the neonatal one (adult: 822 ± 298 pg/mL, n=20, neonatal cells: 19 ± 10 pg/mL, n=18, p < 0.0001). IL-12 or IL-18 did not increase the IFN-γ response of neonatal and adult cells at the used concentrations, while IL-15 increased it by meanly 5-fold (PBMCs) and 40-fold (CBMCs). This suggests a synergistic effect between IL-15 and rTcMIP, particularly on neonatal cells. A higher concentration of IL-18 could also synergize with rTcMIP, but the response in the presence of IL-15 remained the most efficient (**Supplementary Figure 3**). Combinations of low doses of cytokines two by two with rTcMIP did not significantly modify IFN-γ production as compared to cytokines alone (**Figure 2A**). These results pinpoint IL-15 as the most efficient cytokine (amongst the cytokine tested) that synergizes with rTcMIP for IFN-γ production by cord blood and adult mononuclear cells.

### 3.3 rTcMIP can directly activate NK cells

We investigated the effect of rTcMIP on purified cord blood and adult NK cells in the presence or not of added IL-12, IL-15 and/or IL-18. rTcMIP alone did not trigger IFN-γ production by neonatal NK cells and triggered very occasionally a low production of IFN-γ by adult cells (**Figure 2B**). In the presence of IL-15, markedly higher levels of IFN-γ were produced by both cord and adult NK cells. The response of neonatal NK cells increased meanly 60 times and that of adult NK cells around 200 times, reaching levels strongly higher than in supernatants of neonatal NK cells. This indicates that rTcMIP itself can directly activate NK cells provided they are primed/sensitized by IL-15, and that adult NK cells are more sensitive to rTcMIP associated with IL-15 than cord blood NK cells. We also noticed that IL-12 or IL-18, alone or together, did not or hardly elicited IFN-γ release by cord or adult NK cells culture with rTcMIP.

### 3.4 rTcMIP induces expression of TNF-α by cord blood monocytes, dendritic cells and NK cells

**Figure 3:**
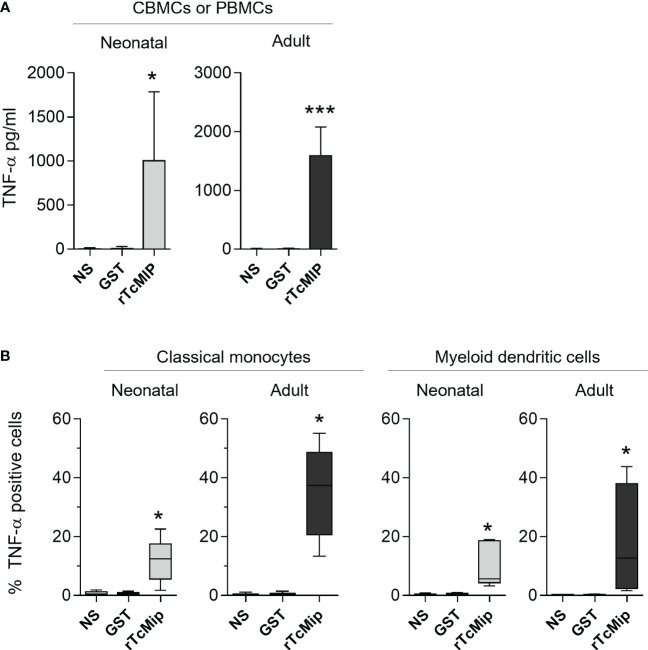
rTcMIP stimulates cord blood and adult blood cells to produce TNF-α. **(A)** CBMCs (n = 5) and PBMCs (n = 20) were stimulated with rTcMIP (2.5 µg/mL) or GST (1.25 µg/mL) as control for 24h. TNF-α released in the supernatants was measured by ELISA. Results are expressed as mean ± SEM **(B)** The frequency of TNF-α expressing cells among CD14^+^CD16^-^ classical monocytes after 4h and CD11c^+^ CD123^-^ myeloid dendritic cells at 24h, was analyzed by flow cytometry (n = 5 in each group). Results are expressed as box-and whisker plots. *: P < = 0.05, ***: P < 0.005 vs. GST (Wilcoxon test). NS: non-stimulated cells.

We investigated the production of TNF-α, as it is an essential mediator of inflammation required for efficient maturation of myeloid cells into mature APCs, also involved in Th1 polarization in association with IFN-γ, and in memory cell formation (20, 21). rTcMIP triggered significant TNF-α release by CBMCs and PBMCs cultured with rTcMIP for 24h (without any addition of exogenous cytokines) (**Figure 3**). Though the response of neonatal cells was lower than the adult one (medians: 292 and 872 pg/mL respectively), the difference was not statistically different. No co-stimulatory cytokines were needed for an efficient production of TNF-α. Flow cytometry investigation showed significant TNF-α expression in response to rTcMIP by neonatal and adult classical CD14^high^ CD16^-^ monocytes after 4h (% of TNF-α positive cells: medians = 12.4% and 37.4% respectively), and after 24h by conventional, myeloid CD11c^+^ dendritic cells (DCs), (22) (% of TNF-α positive cells: medians = 5.61% and 12.7% respectively). The difference between neonatal and adult responses was statistically significant for monocytes (p= 0.008) but not for DCs. Intermediate CD14^+^ CD16^+^ monocytes and plasmacytoid CD11c^-^ CD123^+^ dendritic cells, NK cells and T cells remained negative (**Supplementary Figure 3**).

### 3.5 TNF-α contributes to the IFN-γ response of NK cells

**Figure 4:**
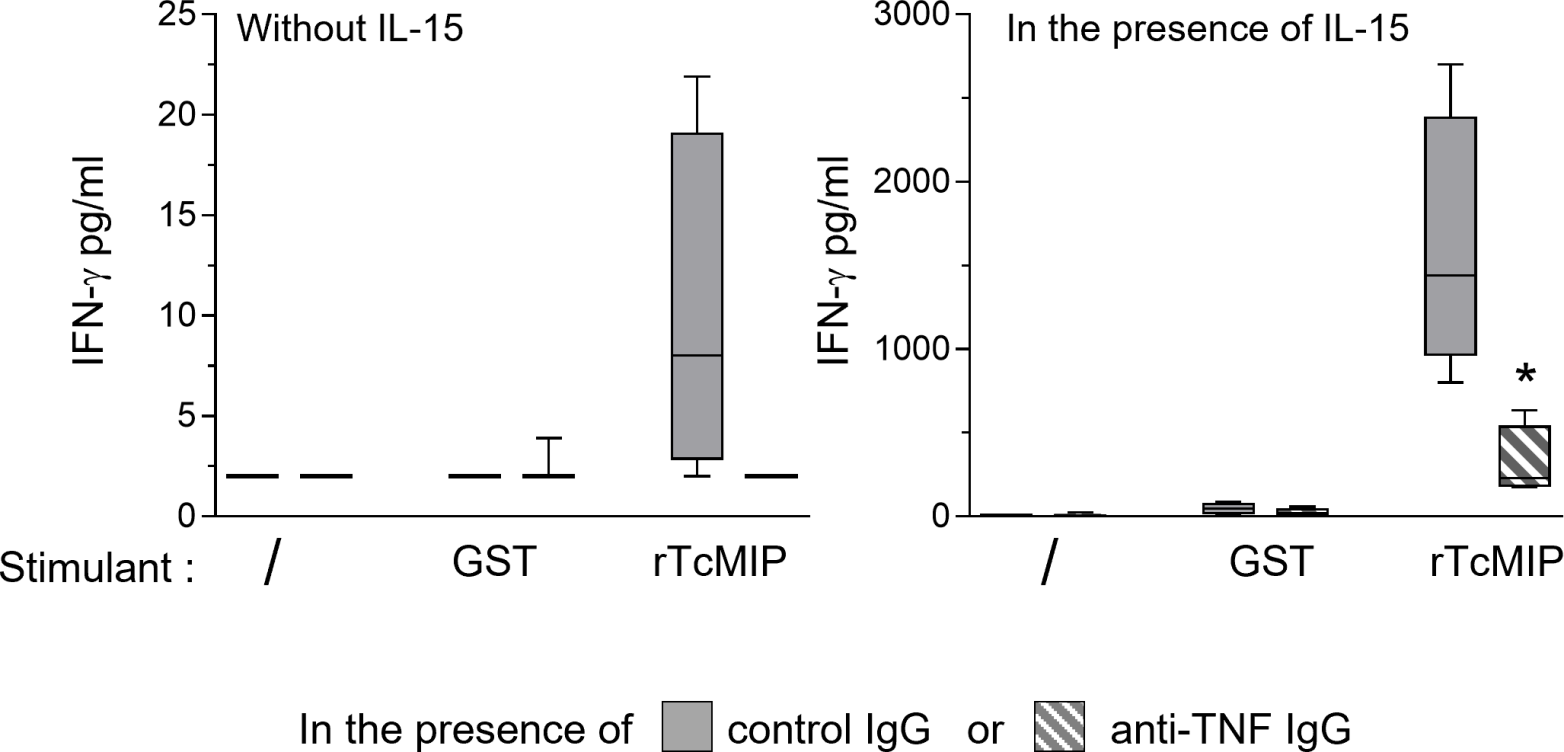
Effect of TNF-α neutralization on the IFN-γ response of CBMCs to rTcMip. CBMCs were cultured during 72h with rTcMip (2.5 µg/mL), GST (1.25 µg/mL) as control, or left unstimulated, in the presence of IL-15 (2.5 ng/mL) and in the presence of TNF-α neutralizing mAb or of control IgG (5 µg/mL). IFN-γ was measured in the supernatants by ELISA. Results are expressed as box-and whisker plots (n=4). *: P < = 0.05 vs. control IgG (Wilcoxon test).

TNF-α has been shown to favor IFN-γ production by NK cells (23). We investigated whether the rTcMIP-induced TNF-α contributed to the IFN-γ response of CBMCs by using TNF-α neutralizing Abs. When cells were cultured in the presence of IL-15, TNF-α neutralization resulted in a significant decrease by meanly 62% of the IFN-γ production by CBMCs (**Figure 4**). In the absence of IL-15, the IFN-γ response was very low (as mentioned above – **Figure 2A**) but nevertheless became undetectable in the presence of the TNF-α neutralizing Abs (data not shown). This indicates that rTcMIP-induced TNF-α contributed to the IFN-γ response of NK cells.

### 3.6 rTcMIP engages TLR2/1 and TLR4

**Figure 5:**
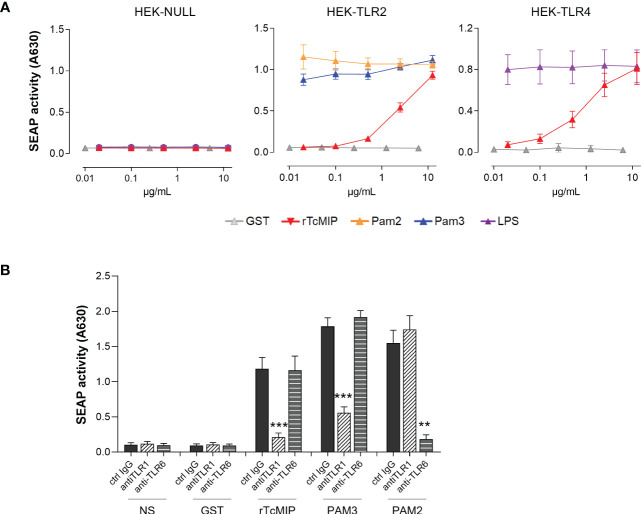
rTcMIP activates TLR2/1 and TLR4. **(A)** HEK-Blue cell line stably transfected by hTLR2 (n = 5) or hTLR4 (n = 5) were stimulated with increasing concentrations of rTcMIP or GST as control. Pam3CSK4 (TLR2/1 agonist), Pam2CSK4 (TLR2/6 agonist) and LPS (TLR4 agonist) were used as positive control for TLR2 and TLR4 activation. HEK-Blue Null cell line (n = 5) transfected with an empty plasmid that does not express neither TLR2 nor TLR4 was used as a control. After 24h incubation, SEAP activity in supernatants was assessed. **(B)** Response of HEK-Blue hTLR2 cells to rTcMIP (2.5 µg/mL) in the presence of anti-TLR1, anti-TLR6 or control IgG (5 µg/mL). The effect of anti-TLR1 and anti-TLR6 mAb on activation by Pam3CSK4 (20 ng/mL) and Pam2CSK4 (0.2 ng/mL) was used as control of the mAb efficacy. Results are expressed as mean ± SEM (n = 5). **: P < = 0.05, ***: P < 0.005 vs. control IgG (Wilcoxon test).

Other pathogens express MIPs, that present a certain degree of homology with rTcMIP (13). Knowing that the MIP of *Chlamydia trachomatis* was shown to interact with TLR2 (24, 25), we investigated the potential implication of TLRs in rTcMIP activity using HEK293 cells with TLR2 and TLR4. Pam3CSK4 (TLR2 agonist) and LPS (TLR4 agonist) were used as positive controls. We found that rTcMIP strikingly activated both cell lines in a dose-dependent manner (**Figure 5A**). rTcMIP did not induce any response by HEK null cells transfected with an empty vector.

TLR2 can recognize ligands mostly in an heterodimeric form with TLR1 and TLR6 (26). Since HEK-Blue hTLR2 cells constitutively express TLR1 and TLR6, we investigated their involvement by using neutralizing mAb. Specific TLR1/2 (Pam3CSK4) and TLR2/6 (Pam2CSK4) ligands were used as controls. Pam3CSK4 and Pam2CSK4 responses were reduced by TLR1 and TLR6-specific mAb respectively, demonstrating their efficacy and specificity at the used concentrations. TLR1 but not TLR6 neutralization significantly tailed away rTcMIP TLR2-dependent activation on HEK-Blue hTLR2 cells (**Figure 5B**).

These results pinpoint TLR2/1 and TLR4 as rTcMIP receptors.

### 3.7 rTcMIP-induced IFN-γ and TNF-α responses of blood cells involve TLR2 and TLR4 independently of CD14

**Figure 6:**
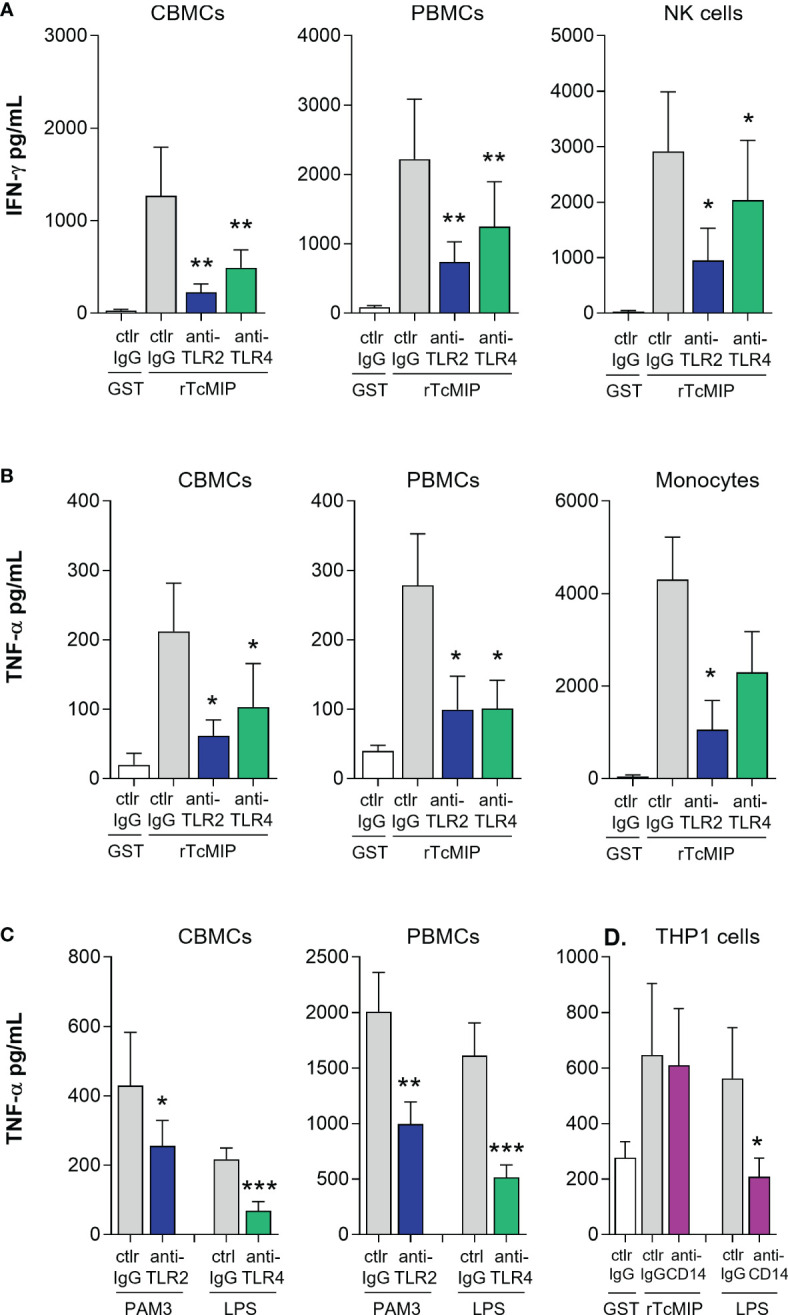
rTcMIP acts through TLR2 and TLR4 independently of CD14. **(A, B)** CBMCs (n = 9 for IFN-γ, 5 for TNF-α), PBMCs (n = 12 for IFN-γ, 5 for TNF-α), purified NK cells (n = 5) and monocytes (n = 5) were stimulated with rTcMip (2.5 µg/mL) in the presence of TLR2 or TLR4 neutralizing mAb or control IgG mAb (5 µg/mL), for 72h in the presence of IL-15 (2.5 ng/mL) for IFN-γ measurement **(A)**, or during 24h without IL-15 for TNF-α measurement **(B)**. **(C)** The neutralizing effects of anti-TLR2 and anti-TLR4 mAb were verified on the response to Pam3CSK4 (TLR2 agonist, 10 µg/mL) and LPS (TLR4 agonist, 0.1 µg/mL). **(D)** PMA-primed THP-1 cells (n = 7 independent experiments) were cultured with rTcMIP (5 µg/mL) or LPS (0.1 µg/mL) for 24h in the presence of anti-CD14 neutralizing mAb or control mAb (5 µg/mL). TNF-α was measured by ELISA in supernatants. Cytokines were measured in supernatants by ELISA. All results are expressed as mean ± SEM. *: P<0.05, **: P < = 0.05, ***: P<0.005 vs. control IgG (Wilcoxon test).

CBMCs, PBMCs or adult purified NK cells and monocytes cultured with rTcMIP were exposed to specific TLR2 and TLR4 blocking antibodies. TNF-α release induced by control TLR ligands (Pam3CSK4 and LPS) was reduced by TLR2 and TLR4-specific mAb respectively, demonstrating their efficacy at the used concentrations. Neutralization of TLR2 or TLR4 significantly reduced the release of IFN-γ and TNF-α by neonatal as well as adult mononuclear cells (**Figure 6A**). Addition of these neutralizing mAb to purified NK cells or monocytes also reduced the release of IFN-γ and TNF-α respectively, indicating that rTcMIP can directly activate NK cells and monocytes via TLR2 and TLR4 (**Figure 6B**).

CD14 is a well know co-receptor of TLR2 and TLR4. This surface GPI-anchored molecule transfers some ligands to TLRs and can help promoting TLR-mediated activation (27, 28). We therefore also investigated its involvement in rTcMIP induced responses using neutralizing mAb. Blockage of CD14 by mAb did not inhibit the ability of rTcMIP to induce TNF-α release, indicating that engagement of TLR2 and TLR4 by rTcMIP is independent of CD14 (**Figure 6C**).

### 3.8 The TNF-α response to rTcMIP involves NF-κB activation through the MyD88-dependent pathway

**Figure 7:**
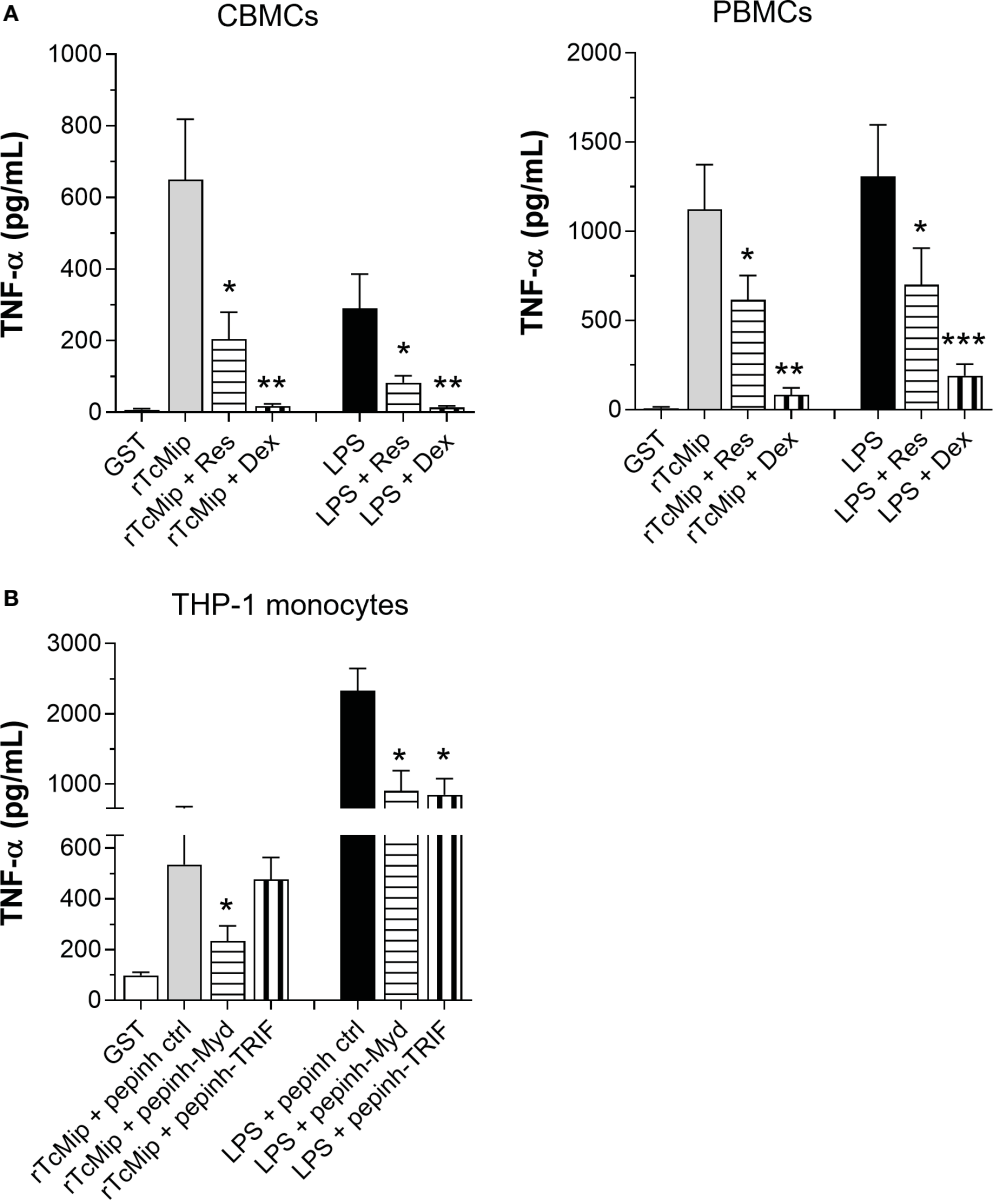
The TNF-α response to rTcMIP depends on MyD88 and NF-kB. **(A)** PMA-primed THP-1 monocyic cells (n = 6 independent experiments) were cultured for 24h with rTcMip (5 µg/mL) in the presence or not of Pepinh-MYD, Pepinh-TRIF or Pepinh control (ctrl) peptides (10 µM). **(B)** CBMCs (n = 5) and PBMCs (n = 5) were cultured with rTcMip (5 µg/mL) in the presence or not of resveratrol (80 µM - Res) or dexamethasone (40 ng/mL – Dex) for 24h. Secreted TNF-α in the supernatants was measured by ELISA. Results are expressed as mean ± SEM. *: P < 0.05, **: P < 0.005, ***:P < 0.001 vs. rTcMIP treated with control pepinh **(A)** or TcMIP alone **(B)** (Wilcoxon test).

NF-κB plays a key role in TLR-induced pro-inflammatory cytokine production (26). We investigated the involvement of NF-κB in the rTcMIP-induced TNF-α response. CBMCs and PBMCs were stimulated with rTcMIP and treated with resveratrol and dexamethasone, two drugs able to prevent NF-κB activation (29, 30). Both strongly inhibited the release of TNF-α by CMBCs and PBMCs (**Figure 7A**). Resveratrol inhibited the CBMC and PBMC responses by meanly 66.6 and 45.6% respectively, the dexamethasone by 94.6 and 92.6% respectively. The effect of LPS was similarly inhibited by both drugs.

TLR2 activates NF-κB by recruiting the adaptor molecule MyD88 while TLR4 can activate NF-κB via MyD88 and TRIF adaptors (26). We investigated if rTcMIP activates both pathways. We treated PMA-primed THP-1 cells with the MyD88 and TRIF specific inhibitory peptides Pepinh-MYD and Pepinh-TRIF prior to the addition of rTcMIP. MyD88 inhibition significantly reduced the TNF-α response to rTcMIP by 35.3% (inhibition of the TRIF pathway did not affected the response (**Figure 7B**). Meanwhile, as expected, both inhibitory peptides inhibited the response to LPS (by 61.3 and 63.7% with Pepinh-MYD and Pepinh-TRIF respectively). We confirmed in mice the essential role of the MyD88 pathway in the response to rTcMIP, where the deletion of the MyD88 gene totally abolished the TNF-α response (**Supplementary Figure 4**).

These results indicate that rTcMIP induces TNF-α production through the MyD88-dependent pathway, leading to NF-κB activation.

### 3.9 FK506 did not affect rTcMIP-induced IFN-γ and TNF-α neither TLR2 nor TLR4 activation

**Figure 8:**
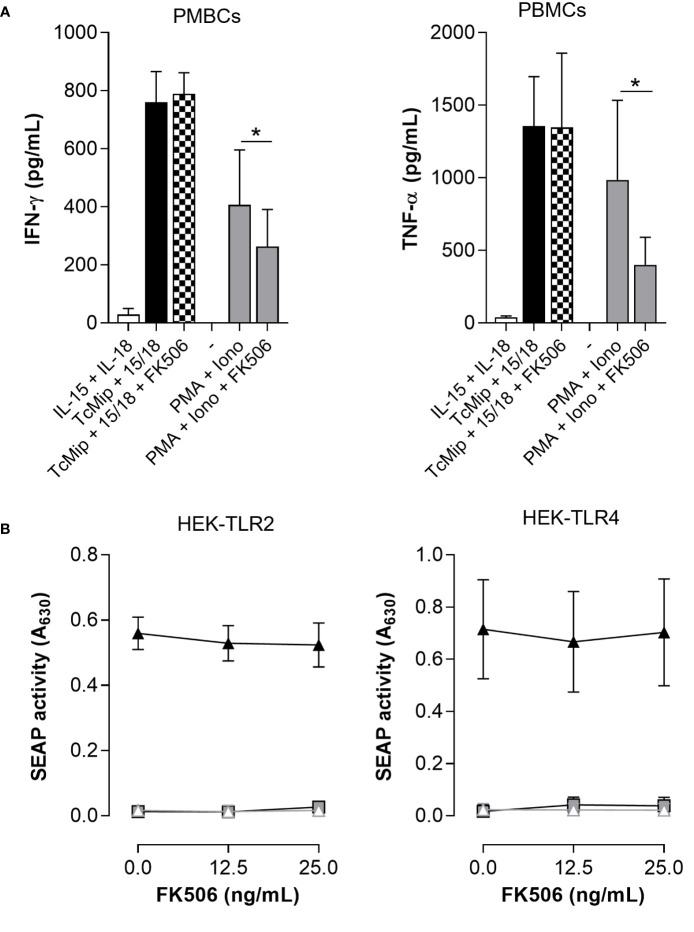
FK506 did not affect TcMIP-induced IFN-γ and TNF-α productions nor TLR2 and TLR4 activation. **(A)** PBMCs (n = 6) were cultured for 72h with rTcMIP (2.5 µg/mL) that was pre-treated or not with FK506 (25 ng/mL). Cultures were performed in the presence of IL-15 (2.5 ng/mL). We controlled the inhibitory potential of the same concentration of FK605 on the response of cells stimulated with PMA (1 µg/mL) and ionomycin (10 µg/mL). IFN-γ and TNF-α were measured by ELISA in supernatants. Results are expressed as mean ± SEM, *: P<0.05 vs. the absence of FK506 (Wilcoxon test). **(B)** Similarly, HEK-Blue hTLR2 and HEK-Blue hTLR4 cells were stimulated with rTcMIP (2.5 µg/mL) pre-incubated with increasing amounts of FK506 (0, 12.5 and 25 ng/mL). Results are expressed as mean ± SEM of 4 independent experiments.

rTcMIP belongs to the family of FK506 binding proteins (FKBPs) (12). Such proteins are peptidyl-prolyl cis-trans isomerases (PPIase), and this activity can be inhibited by the macrolide antibiotic FK506 (31). To investigate if the immunostimulatory activity of rTcMIP was associated with its PPIase activity, FK506 was added to the culture of mononuclear cells stimulated by rTcMIP. We used cells stimulated by PMA and ionomycin as control of the inhibitory effect of FK506 (32). **Figure 8A** shows that FK506 did not affect the rTcMIP ability to induce IFN-γ and TNF-α release, while the response to PMA and ionomycine was significantly inhibited by 57.4 ± 13.6%. Besides, to study whether FK506 could affect TLR2 and TLR4 activation by rTcMIP, we cultured HEK-Blue hTLR2 and HEK-Blue hTLR4 cells with rTcMIP and increasing amounts of FK506. **Figure 8B** shows no significant difference in TLR2 neither TLR4 activation by rTcMIP after FK506 treatment. These observations strongly suggest that TLR2 and TLR4 activation by rTcMIP and the ensuing IFN-γ and TNF-α responses do not depend on its PPIase enzymatic activity.

## 4 Discussion

We previously identified the immunostimulatory potential of the macrophage infectivity potentiator of the protozoan parasite *Trypanosoma cruzi* (rTcMIP), which induces the release of IFN-γ by cord and adult blood cells (11). IFN-γ is a key cytokine to direct, with IL-12, the differentiation of Th1 CD4+ T cells and CD8+ T cells (33). This let us consider rTcMIP as a potential pro-type 1 adjuvant that might be associated to vaccines, particularly to pediatric vaccines since the immune system in early life is skewed against type 1 responses (4). Vaccine adjuvants act mostly by engaging components of the innate immune system (34, 35). The present study, based on experiments performed with cord and adult blood cells, brings some light on the mechanisms by which rTcMIP activates innate immune responses. We found that rTcMIP engaged TLR1/2 and TLR4 independently of CD14 and activated the MyD88 but not the TRIF signaling pathway. rTcMIP induced, through these receptors, the release of IFN-γ by NK cells and of TNF-α by monocytes and myeloid dendritic cells. The NK-cell co-stimulatory cytokine IL-15 greatly increased their IFN-γ response to rTcMIP. Neutralization experiments showed that TNF-α could also boost IFN-γ expression, though it was not mandatory.

rTcMIP is a peptidyl-prolyl cis-trans isomerase (PPIase) belonging to the FK506 binding protein (FKBP) family of proteins (12). Our results indicated that the engagement of TLR2 and TLR4 by rTcMIP and the IFN-γ and TNF-α responses of PBMCs were independent of its enzymatic site. This is consistent with diverse cellular functions exerted by other FKBPs which are also independent of the PPIase activity (31, 36). Some bacteria also express MIPs, that present 26 to 38% sequence homology with rTcMIP (12, 13). To our knowledge, the only other MIP having been shown to bind a TLR is the MIP of *Chlamydia trachomatis* (24, 37). Both MIPs engaged TLR2 associated with TLR1 and activated the MyD88 pathway while contrary to rTcMIP, the MIP of *Chlamydia* did not engage TLR4. The MIP of *Chlamydia* is a lipoprotein, and its TLR-dependent bioactivity was shown to rely on its lipid moiety. At odds, our recombinant rTcMIP is a pure protein without any post-translational additives (11), except it is bound to a GST tag. We cannot exclude that this tag affects somehow the binding of rTcMIP to TLRs or its bioactivity in an unknown way (38). Nevertheless, GST alone did not display any rTcMIP-like bioactivity. We may be surprised that a pure protein can engage TLR2 and TLR4 as most ligands reported to bind these receptors are lipidated components. Proteinic TLR2 and TLR4 bacterial ligands have however been identified [reviewed in (39)]. A common feature of TLR2 ligands is to be amphiphilic (40). In line with this, rTcMIP possesses a rather large hydrophobic area which is, interestingly, exposed at its surface (13) and might perhaps contribute to its binding to TLR2 and TLR4.

Another difference between rTcMIP and the MIP of *Chlamydia* concerns the involvement of CD14, which was required as TLR2 co-receptor by the MIP of *Chlamydia* but not by rTcMIP (24). CD14 is a surface glycoprotein expressed by monocyte/macrophages. It can bind a wide range of microbial ligands and acts as an adaptor or a co-receptor for several TLRs, including TLR2/1 and TLR4 (41). CD14 can have multiple effects, from only increasing the affinity of a ligand for a TLR to being mandatory for the TLR response, depending on the ligand, the TLR itself and the cell type (28, 42). TLR2 and TLR4 are expressed at the plasma membrane and initially transduce a signal from the cell surface *via* MyD88. Both may be endocytosed and transduce signals from the endosomal compartment. Importantly CD14 plays a central role in driving the endocytosis of these TLRs and the ensuing second wave of signaling (43, 44). This second wave, which remains Myd88-dependent for TLR2 but switches to the TRIF pathway for TLR4, can strengthen the expression of inflammatory cytokines (via NF-κB) as well as induce type I IFNs expression (through IRF3 and IRF7) and IL-10 production (28, 43–48). Since rTcMIP seems to not engage CD14 we may infer that it will not trigger TRIF signaling. This is further supported by the following observations. First, specific inhibition of TRIF did not affect the production of TNF-α meanwhile the deletion of MyD88 abolished TNF-α release. Secondly, we did not detect type I IFNs by ELISA in supernatants of blood cells stimulated with rTcMIP (results not shown). Thirdly dexamethasone inhibited nearly totally the TNF-α response. Dexamethasone is reported to exert an inhibitory effect only if the response does not result from the concomitant activation of the MyD88 and the TRIF pathways (49), supporting that the TNF response resulted from only one of these pathways.

IFN-γ was predominantly produced by the CD56^bright^ subset of NK cells. Although both CD56^bright^ and CD56^dim^ NK cells constitutively contain IFN-γ transcripts (50), this is not surprising since CD56^bright^ NK cells produce IFN-γ preferentially in the presence of co-stimulatory cytokines while the CD56^dim^ subset responds rather to other activating receptors (51). CD56^bright^ NK cells generally require at least two signals to produce IFN-γ. Hence, in our conditions, we assume that one signal is delivered by rTcMIP, another by cytokines that we exogenously added in CBMCs/PBMCs cultures. Several cytokines are able to co-activate NK cells (52). We found that IL-15 was the best one to work together with rTcMIP, over IL-12 and IL-18, at least at the concentrations we used. Of note, at these concentrations, they did not alone induced IFN-γ production by neonatal or adult CD56^bright^ NK cells while rTcMIP did, although faintly in cord blood cells. There was a synergistic effect between IL-15 and rTcMIP, particularly pronounced with neonatal cells.

**Figure 9:**
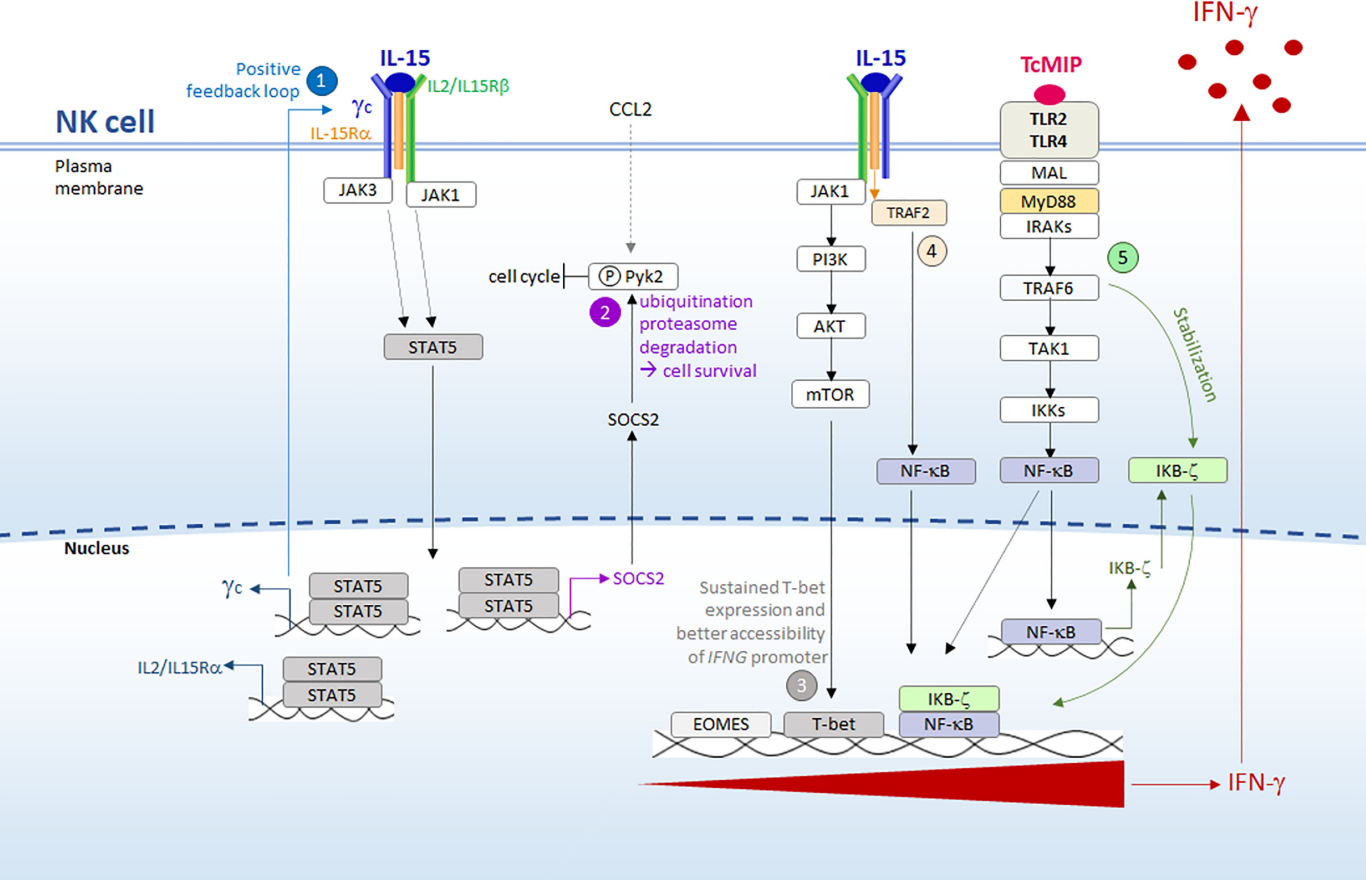
Synergy between IL-15 and rTcMIP for IFN- γ secretion by purified NK cells. We propose the following hypothesis, based on our results and the literature (see text for references): 1) resting NK cells are initially primed/activated by IL-15 (free IL-15 that we exogenously added, or IL-15 that might be produced by local macrophages ref. The ensuing activation of STAT5 enhances the expression the IL-15R, allowing higher signal transduction by a positive feedback loop. 2) STAT5 activation concomitantly favors survival of NK cells by inducing SCOS2 expression, which in human NK cells directs the degradation of phosphorylated Pyk2, a protein that inhibits the cell cycle progression. Pyk2 phosphorylation may derive from signals given by chemokines, such as CCL2 triggered by rTcMIP ou autophosphorylation ref. 3) Through the PI3K/AKT/mTOR pathway (ref) IL-15 leads to sustained T-bet expression and favors the accessibility of *IFNG* promoter to this transcription factor. 4) Meanwhile, NF-kB is activated by both IL-15 and rTcMIP. However, binding of NF-kB to *IFNG* promoter occurs only in the presence of stable expression of the transcription co-activator IKBζ. Whereas transcription of the gene coding for IKBζ is transiently induced by NF-kB, its stable expression is driven by a signal delivered by the MyD88 pathway but independent of NF-kB, signals that in our model can only be delivered by rTcMIP (5). T-bet and NF-kB then synergize to induce IFN-γ expression.

Optimal NK cell production of IFN-γ requires co-stimulation by more than one signal (53). How would rTcMIP and IL-15 work together to induce IFN-γ expression by CD56^bright^ NK cells? Based on the literature and our results, we propose the following hypothesis (**Figure 9**). IL-15 is a critical cytokine for NK cell activation (54). Resting NK cells express the three chains of its high affinity receptor, IL-15Ra, IL-2/15Rβ (CD122) and the γ_χ_ chain (CD132) and are very quickly primed by free IL-15 (54, 55). The ensuing activation of the transcription factor (TF) STAT5 enhances the expression the γ_χ_ and the IL-15Ra chains, allowing higher signal transduction by a positive feedback loop (55) (**Figure 9**, point 1). IL-15 favors survival of NK cells by inducing SCOS2 expression, which in humans directs ubiquitination and proteasomal degradation of phosphorylated Pyk2, a protein which can auto-phosphorylate following homotypic interactions between CD56 expressed by NK cells (56, 57) and which inhibits cell cycle progression and survival (58, 59) (**Figure 9**, point 2). IL-15 also allows sustained expression of T-bet meanwhile favoring the accessibility of the *IFNG* locus to T-bet (**Figure 9** point 3) (51, 60–64), and may possibly contribute to NF-κB activation (**Figure 9** point 4) (65). Together, this might prime NK cells for IFN-γ expression. Beside, IL-15 can endow NK cells to respond to IL-12 and IL-18 by increasing the expression of their receptors (66). However, in our experiments, rTcMIP could induce IFN-γ in synergy with IL-15 by purified NK cells in the absence of exogenous IL-12 or IL-18. As such, we may suppose that IL-15 mainly promoted NK cell survival and contributed to T-bet and NF-κB-dependent priming for IFN-γ expression, without the involvement of IL-12 and IL-18.

What could be the role of rTcMIP in its synergy with IL-15? An attractive hypothesis relies on the mandatory requirement of the protein IKBζ for IFN-γ expression by NK cells. This nuclear protein is a transcriptional coactivator that binds to the promoter region of *IFNG*, allowing the binding of STAT4 and NF-κB, which cooperate to trigger *IFNG* transcription. In the absence of IKB-ζ, these TFs do not bind (67, 68). A stable expression of IKBζ is necessary in order it exerts this positive influence. Stable expression of IKBζ requires two signaling events: one leading to activation of NF-κB, which drives transient expression of IKBζ, and another, which is independent of NF-κB but depends on the MyD88 pathway, which stabilizes IKBζ transcripts (69, 70). rTcMIP may fulfill these two requirements since we showed that it induced signaling trough MyD88 and activated NF-κB (**Figure 9** point 5). Moreover, by triggering CCL2 release (11), rTcMIP might contribute to NK cell survival since CCL2 induces Pyk2 phosphorylation (71), which thereby becomes the target of IL-15-driven SOCS2 (cf. supra). In summary, the synergy between IL-15 and rTcMIP for IFN-γ expression by NK cells may encompass priming and signals for sustained expression of T-bet given by a low dose of IL-15, signals leading to NK cell survival (given by IL-15, and indirectly by rTcMIP via CCL2) and to NF-κB activation (provided by IL-15 and rTcMIP), and signals driving stable expression of IKBζ which allows binding of NF-κB to *IFNG* promoter (provided by rTcMIP). This leads to a synergy between T-bet and NF-κB resulting in IFN-γ expression (**Figure 9**).

In contrast to IL-15, IL-12 and IL-18 used at low concentrations did not potentiate the stimulating action of rTcMIP on NK cells neither significantly increased the IFN-γ response of rTcMIP associated to IL-15. IL-12 is largely reported as crucial for IFN-γ expression by NK cells (52, 72). Yet, IL-12-independent mechanisms of NK cell activation have been reported (73). Our experiments with purified NK cells suggest that IL-12 was not mandatory in our model. This does not discard the possibility that, in mononuclear cell cultures, IL-12 was produced endogenously by DCs or monocytes (74). We could not detect IL-12 in supernatants of PBMCs stimulated during 24h with rTcMIP and IL-15 or of PMA-primed THP1 cells stimulated during 24h with rTcMIP, even with the sensitive assay using HEK293-IL-12 reporting cells (InvivoGen) (unpublished results), arguing against an endogenous production of IL-12. It should however deserves further investigation, knowing that IL-12 may be very difficult to detect as it is produced in very low amounts and confined to immune synapses (75). The lack of effect of IL-18 on the IFN-γ response to rTcMIP likely relies to the fact that this cytokine acts mainly at a post-transcriptional level by stabilizing the *IFNG* transcripts (76), which seems not sufficient to increase the low *IFNG* expression induced by rTcMIP alone.

rTcMIP also induced TNF-α expression by neonatal and adult cells. TNF-α was mainly produced by classical monocytes and myeloid (or conventional) dendritic cells. In view of the dominance of the cDC2 subset amongst CD11c+ myeloid DCs (77, 78), we may suppose that this subset contributes to the TNF-α response to rTcMIP. These cell types are known to highly express TLR2/1 and TLR4 and to be good producers of TNF-α (21, 77). The regulation of TNF-α expression is complex. TNF-α transcription depends on several TFs coordinately activated by the TLR/MyD88 pathway, comprising NF-κB (44). However its expression is essentially regulated post-transcriptionally, mainly through a pathway initiated by p38MAPKs (79). In addition, TNF-α is first produced as transmembrane trimers at the plasma membrane, that have to be cleaved by the metalloprotease ADAM17 to be released (21). Many signals can activate ADAM17 (80), including p38MAPKs (81). The TLRs/MyD88 pathway activates NF-B and p38MAPKs (44), supporting that TNF-α expression by monocytes and cDC2 in response to rTcMIP likely relies on its ability to engage TLR2 and/or TLR4. Although TNF-α is not usually cited as a NK cell co-activating cytokine (18), it activates NF-κB involved in *IFNG* expression (82) and has been shown to augment the IFN-γ response of NK cells stimulated by a combination of IL-2 and IL-12 (23). In line with this, our results indicate that TNF-α acted as a significant co-stimulatory cytokine for the IFN-γ response of NK cells to rTcMIP, used alone or associated with IL-15. We may speculate that rTcMIP activates monocytes and cDC2 to produce TNF-α that, in combination with a direct effect of rTcMIP on NK cells, licenses them to produce IFN-γ. However, TNF-α was dispensable since purified NK cells, which did not express TNF-α in our conditions, produced IFN-γ in response to rTcMIP (combined with IL-15).

TNF-α can act through two receptors. TNFR1 signaling is activated by both soluble and membrane TNF while TNFR2 is reported to be activated primarily by membrane TNF (82). As NK cells express mainly the TFNR2 (23), it implies that the action of TNF-α on NK cells would rely mostly on membrane TNF-α expressed by monocytes and/or DCs (82). This suggests that contacts between NK cells and monocytes and/or DCs occurs. It might explain why rTcMIP alone was not able to induce IFN-γ by purified NK cells while it did (though at low levels) in CBMCs/PBMCs cultures.

Together, our results indicate that besides accessory cells, rTcMIP exerts a direct action on NK cells. The stronger inhibitory effect of TLR2 over TLR4 neutralization suggests that NK cells preferentially responded to TLR2. In line with this, it has been reported that direct TLR2 signaling may be critical for NK cell activation (83).

The early inflammatory environment considerably influences the orientation of the adaptive immune response as well as its memory potential, which are of upmost importance for vaccine efficacy (9, 84). TLRs are considered as excellent targets for adjuvants to provide danger signals that induce an immune response leading to long-lasting protection (85). We therefore speculate that rTcMIP, through its ability to trigger TLR-dependent innate IFN-γ and TNF-α production and to activate innate cells such as NK cells, monocytes and cDCs, might be an interesting vaccine adjuvant molecule. TNF-α is a highly pleiotropic cytokine that, among other functions, plays an important role in the maturation of APCs licensed to activate T cells (21). This effect of TNF-α relies on its ability to induce NF-κB. We may assume that rTcMIP, by binding TLR2 and TLR4 expressed by cDCs, could reinforce the effect of TNF. rTcMIP also activates classical monocytes to produce TNF-α. Classical monocytes are reported to be prone to infiltrate inflammatory sites as well as to differentiate into monocyte-derived DCs (86). It is possible that rTcMIP would also activate macrophages present at the site of vaccine administration. This would create a micro-environment that, alongside with IFN-γ released by NK cells in response to rTcMIP, might upregulate monocytes/macrophages IL-15 expression (87, 88) and maybe also IL-12. Moreover, CD56^bright^ NK cells are reported to migrate to lymph nodes (89) where, by producing IFN-γ, they will contribute to drive a type 1 immune response characterized by the activation of Th1 CD4+ T cells, CD8+ T cells and the production of type 1 Ab isotypes (90). In addition, TNF-α favors Th1 response in association with IFN-γ (20, 91). Altogether, our results argue for a pro-type 1 effect of rTcMIP, in agreement with our previous results in mouse models of vaccination with ovalbumin and pertussis, where Abs of IgG2a isotype, witnessing of IFN-γ skewing (92), were preferentially produced (11).

An important requirement for vaccine efficacy against intracellular pathogens is to trigger a long-lasting T memory immune response. Interestingly, activated tissue monocytes have recently been disclosed to play a key role in inducing the formation of tissue-resident memory CD4^+^ and CD8^+^ T cells (93). If rTcMIP would endow monocytes to achieve this, merits to be investigated. The induction of innate IFN-γ by adjuvants has anyway been reported as central to drive protective immune responses to vaccines (94), emphasizing again that rTcMIP might be a promising vaccine adjuvant.

Interestingly, rTcMIP activated cord blood innate cells. Though the neonatal responses of NK cells and monocytes were globally lower than the adult ones, our previous experiments in neonatal mice supported the adjuvant property of rTcMIP (11). It might even be advantageous to induce a type 1 immune response while not triggering a too strong inflammatory response, since high inflammation is deleterious in early life (95, 96), which is one of the reasons why the neonatal immune system physiologically moderates inflammation and is skewed towards preferentially mounting Th2 and regulatory T cell responses (4).

Nonetheless, if our work pinpoints rTcMIP as a potential efficient adjuvant for pediatric vaccines, huge work has still to be done. The mechanism of action should be further investigated with high-throughput approaches in human *in vitro* systems vaccinology (97–99). It should also be examined if rTcMIP would induce cross-antibodies to human FKBPs. Indeed, rTcMIP is a FKBP presenting homology with human FKBP12 (13). Interestingly, the exposed hydrophobic part of rTcMIP, which we hypothesize to be involved in its binding to TLR2 and TLR4 (cf. supra), is absent in human FKBP12 (13). Investigating the relation between the immunostimulatory properties of rTcMIP with its structure should perhaps allow identifying a rTcMIP-specific fragment that could avoid the problematic of cross-antibodies with human FKBP. Another drawback is that a protein would induce antibodies against itself, which might limit its repeated administration. However, this handicap might turn into a benefit. rTcMIP was first described for its role as promoting invasiveness of the parasite into host cells (12). If neutralizing Abs would be induced, it might perhaps afford some protection against Chagas disease and even against other MIP-expressing pathogens. In line with this, MIPs expressed by several pathogenic bacteria have these recent years been identified as potential vaccine antigens (36). Another advantage of rTcMIP “side-effects” such as cross-Abs, together with activation of innate cells, might offer a “pathogen-agnostic” protection in the neonatal period, i.e. might boost resistance through non–pathogen specific mechanisms (100).

Finally, we would like to point out that the engagement of two different TLRs by rTcMIP might be a benefit for its use as adjuvant associated with sub-unit vaccines (both peptide- or nucleic acid-based) (101). Indeed, the concomitant activation of multiple innate receptors accounts for the better efficiency of live attenuated vaccines in inducing protective immune responses. A such, it has been reported that TLR2 agonists enhance T cell and antibody responses without altering the Th1/Th2 cell balance, while TLR4 agonists drive polarized Th1 cell responses (34). On the other hand, the fact that rTcMIP is a recombinant protein opens the possibility to use it in “built-in, self adjuvanting” vaccines, which may present several advantages such as ensuring that the APCs activated by the adjuvant are the same APCs exposed to antigen (34, 102, 103). Moreover, Mc Donald et al. report that an amphiphilic self-adjuvanting vaccine, by giving rise to multiple order structures, improves vaccine efficacy by enabling multimeric antigen presentation. Interestingly, the macrophage infectivity potentiator of *T. cruzi* is an amphiphilic protein (13), a property that hence might be an advantage. In line with this, analysis of our rTcMIP fusion protein by native PAGE indicated it indeed forms aggregates in physiological conditions (unpublished result).

## Data availability statement

The raw data supporting the conclusions of this article will be made available by the authors, without undue reservation.

## Ethics statement

The studies involving human participants were reviewed and approved by Ethical Committee Erasme-U.L.B. of the Faculty of Medicine, U.L.B. (protocols P2011-254 and P2014-339). Written informed consent to participate in this study was provided by the participants’ legal guardian/next of kin. The animal study was reviewed and approved by Institutional Animal Welfare Committeeof Sciensano (file numbers 20161007–01 and20180913–01).

## Author contributions

Conceptualization: CT, SAD Funding acquisition: CT Investigation: all authors. Data analysis: all authors Writing–original draft: CT Writing–review and editing: SAD, NS, CT. All authors contributed to the article and approved the submitted version.
