# Supplementary figures and images for: The macrophage infectivity potentiator of Trypanosoma cruzi induces innate IFN-γ and TNF-α production by human neonatal and adult blood cells through TLR2/1 and TLR4
Source: Front Immunol. 2023 May 26;14:1180900. doi: 10.3389/fimmu.2023.1180900 (PMC10250606; doi:10.3389/fimmu.2023.1180900)

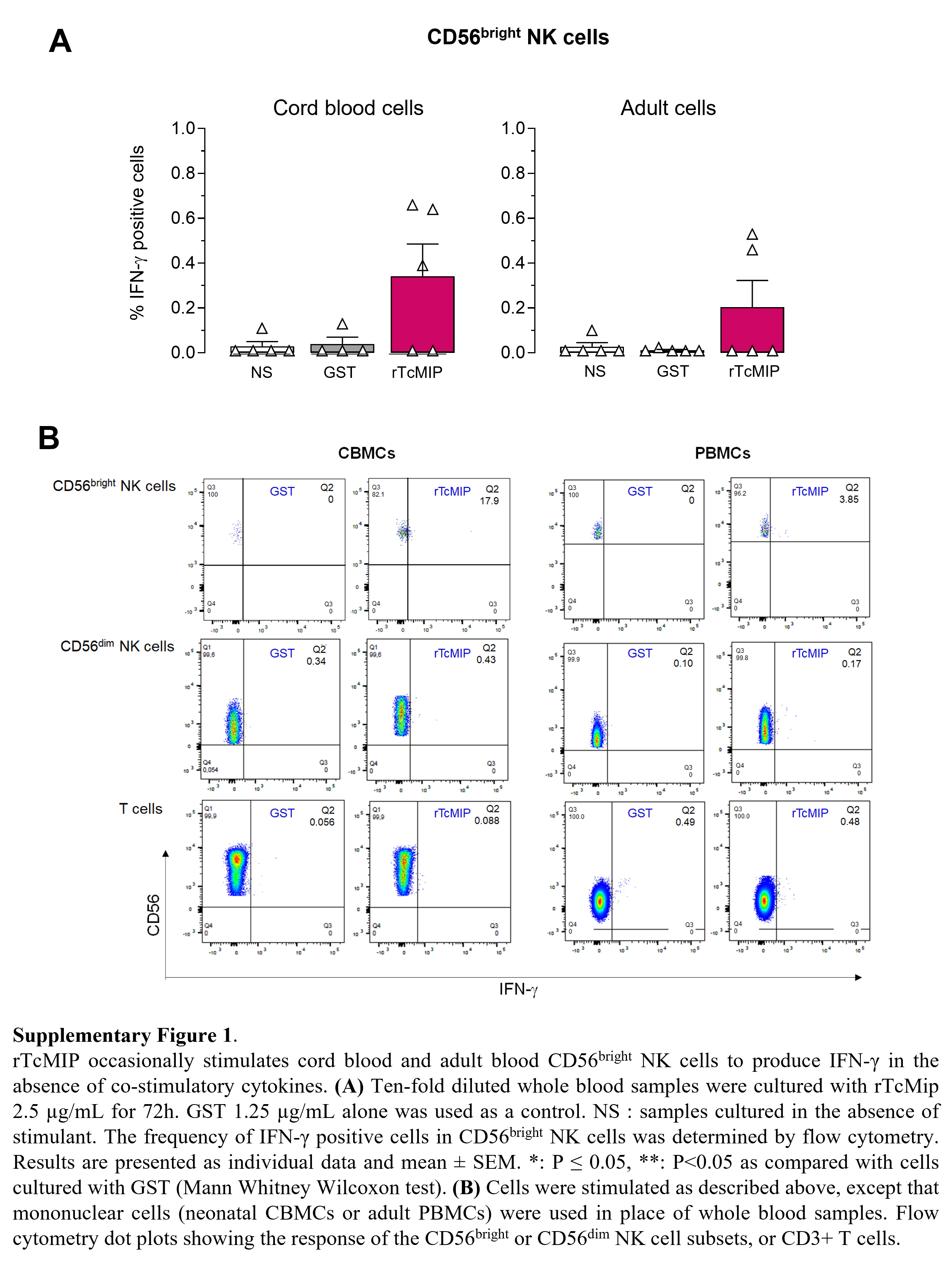

Supplement: Supplementary file 1 [file Image_1.tif]

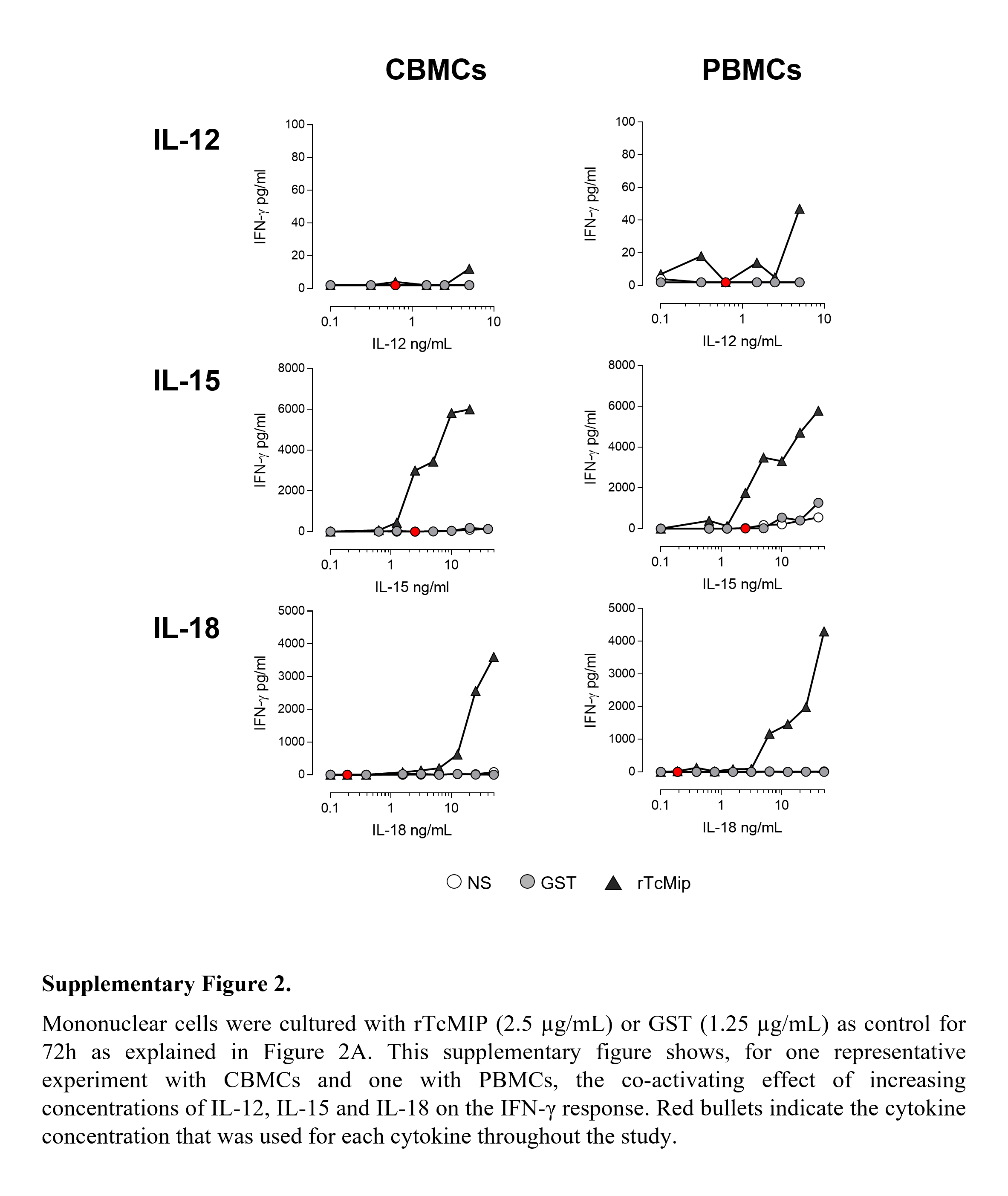

Supplement: Supplementary file 2 [file Image_2.tif]

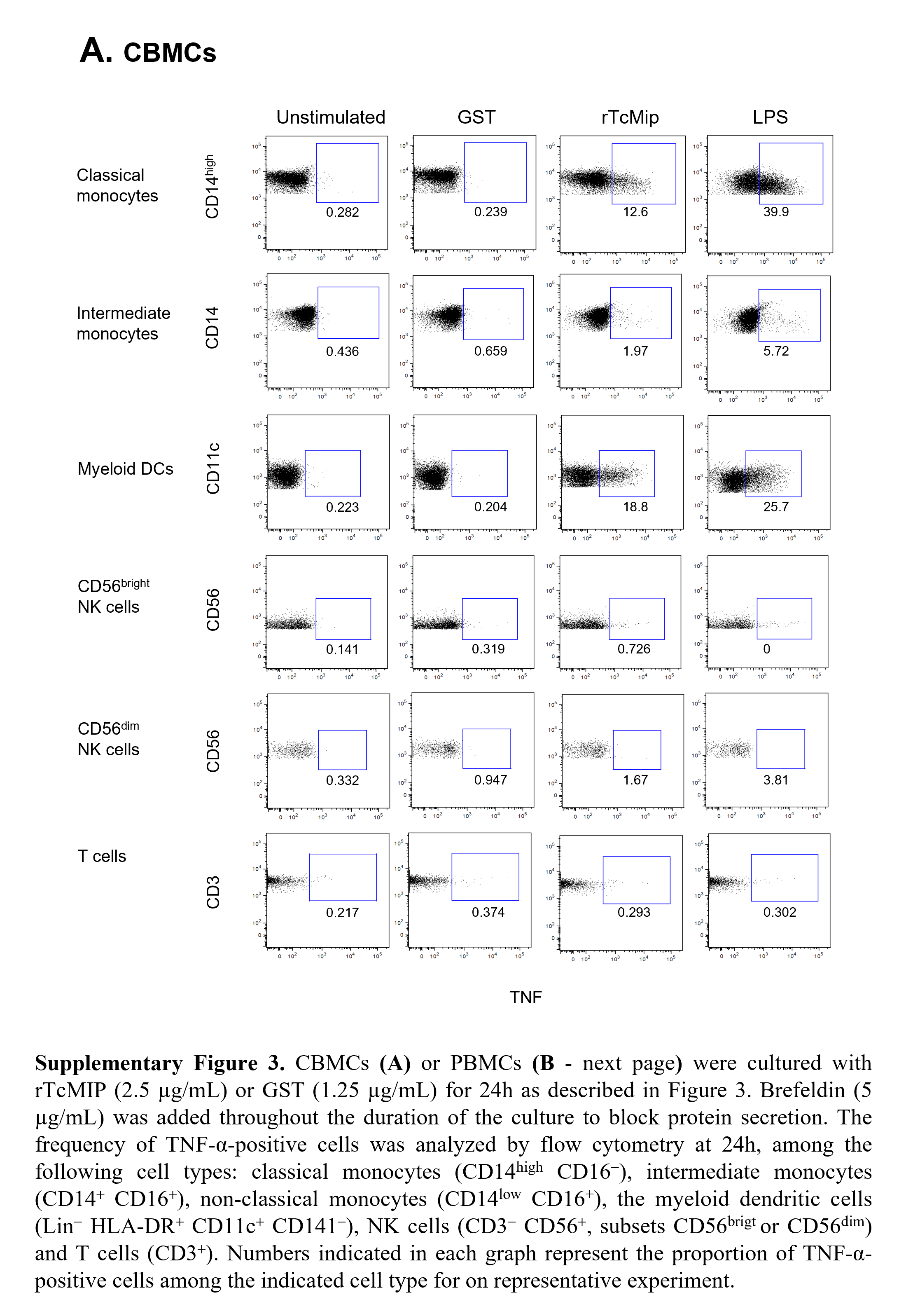

Supplement: Supplementary file 3 [file Image_3.tif]

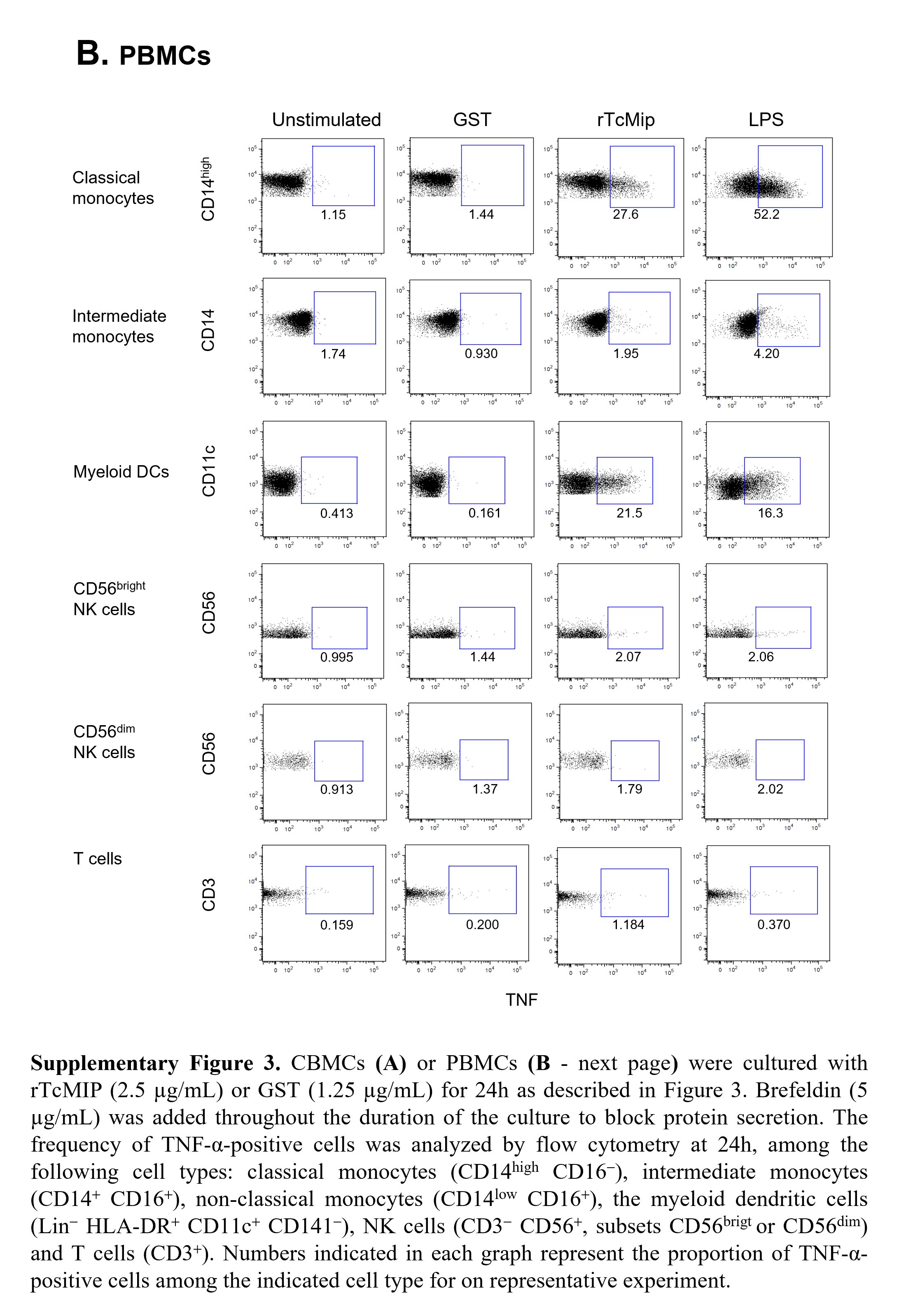

Supplement: Supplementary file 4 [file Image_4.tif]

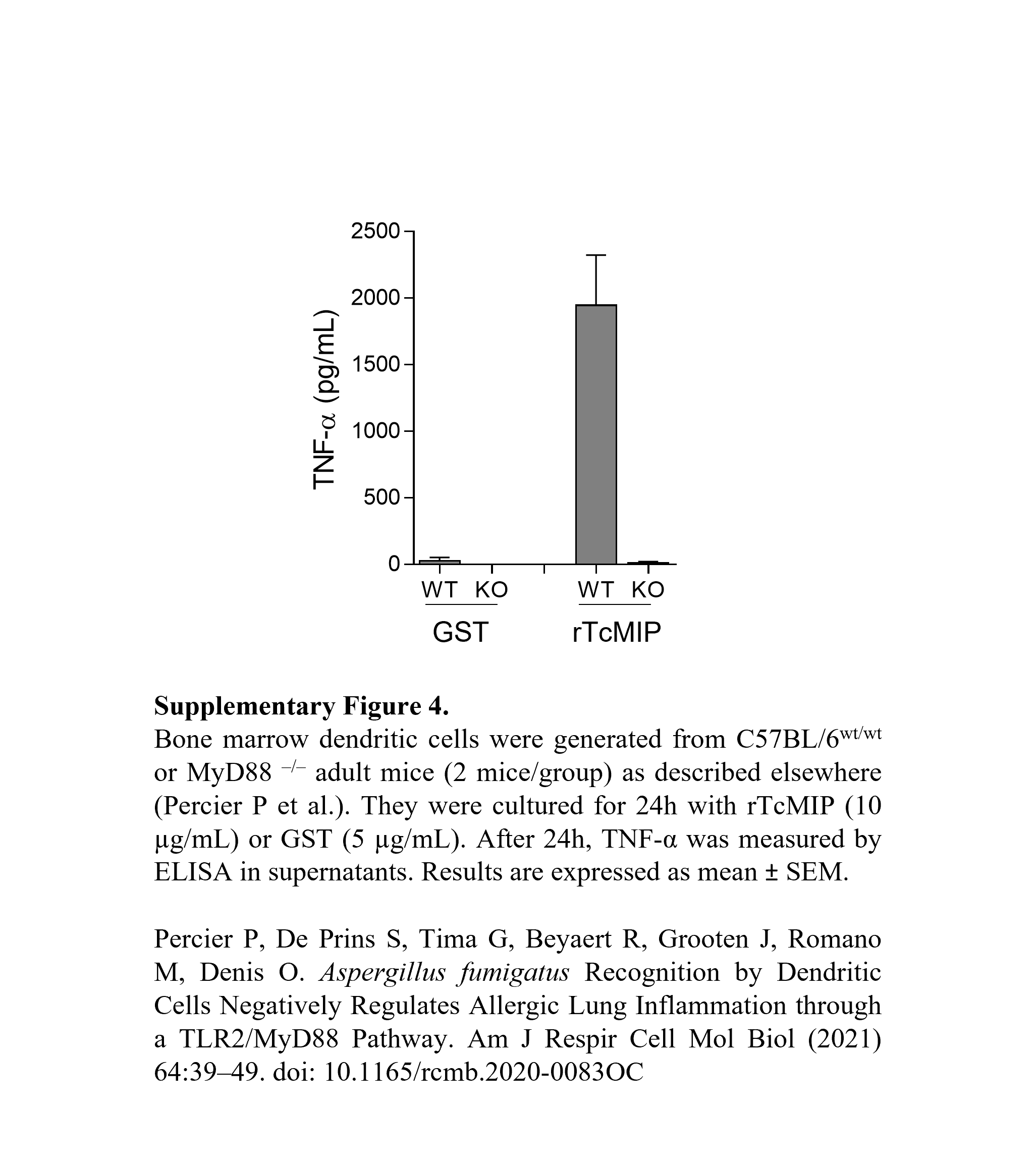

Supplement: Supplementary file 5 [file Image_5.tif]
